# Trauma and post-traumatic stress disorder in patients treated for opioid use disorder: findings from a 12-month cohort study

**DOI:** 10.1192/bjo.2021.971

**Published:** 2021-07-22

**Authors:** Tea Rosic, Vivian Y. O. Au, Andrew Worster, David C. Marsh, Lehana Thabane, Zainab Samaan

**Affiliations:** Department of Psychiatry and Behavioral Neurosciences, McMaster University, Ontario, Canada; and Department of Health Research Methods, Evidence, and Impact, McMaster University, Ontario, Canada; Michael G. DeGroote School of Medicine, McMaster University, Ontario, Canada; Department of Health Research Methods, Evidence, and Impact, McMaster University, Ontario, Canada; and Department of Medicine, McMaster University, Ontario, Canada; Northern Ontario School of Medicine, Ontario, Canada; Canadian Addiction Treatment Centres, Ontario, Canada; and ICES North, Ontario, Canada; Department of Health Research Methods, Evidence, and Impact, McMaster University, Ontario, Canada; Biostatistics Unit, Research Institute at St Joseph's Healthcare, Ontario, Canada; Department of Pediatrics, McMaster University, Ontario, Canada; and Department of Anesthesia, McMaster University, Ontario, Canada; Department of Psychiatry and Behavioral Neurosciences, McMaster University, Ontario, Canada; and Department of Health Research Methods, Evidence, and Impact, McMaster University, Ontario, Canada

**Keywords:** Addiction, opioid use disorder, post-traumatic stress disorder, comorbidity, opioid agonist therapy

## Abstract

**Background:**

Exposure to traumatic events is both a risk factor for substance use and an adverse outcome of substance use disorders. Identifying and managing post-traumatic stress disorder (PTSD) in patients with addiction requires attention.

**Aims:**

To examine the lifetime prevalence of traumatic events and past-month prevalence of PSTD in patients treated for opioid use disorder, and explore the association between trauma, PTSD and treatment outcomes.

**Method:**

Participants (*n* = 674) receiving methadone treatment in 20 community clinics across Ontario, Canada, were administered the Mini-International Neuropsychiatric Interview to identify self-reported traumatic events and PTSD. Drug use was measured for 12 months by urine drug screens.

**Results:**

Eleven per cent of participants met past-month criteria for PTSD (*n* = 72), and 48% reported history of traumatic events with no current PTSD (*n* = 323). Participants with PTSD were more likely to be female (odds ratio 2.13, 95% CI 1.20–3.76) and less likely to be employed (odds ratio 0.31, 95% CI 0.16–0.61) or married (odds ratio 0.51, 95% CI 0.26–0.90) than those with no trauma history. Antidepressants (39 *v*. 24%) and benzodiazepines (36 *v*. 18%) were differentially prescribed to patients with and without PTSD. Length of time in treatment and opioid use were not associated with trauma; however, suicidal ideation was more common in PTSD (odds ratio 2.29, 95% CI 1.04–5.01).

**Conclusions:**

Trauma and PTSD are prevalent among patients with opioid use disorder, and consideration of trauma symptoms and associated characteristics is warranted. Patients with and without comorbid PTSD differ clinically and psychosocially, highlighting the relevance of integrating addiction and mental health services for this population.

Exposure to traumatic events such as threatened death or serious injury, and the experience of trauma (the emotional response to a traumatic event), have been associated with the development of substance use disorders (SUDs).^1^ Furthermore, individuals with SUDs may be at greater risk of experiencing violence and traumatic events.^2^ Not all individuals who experience trauma develop post-traumatic stress disorder (PTSD); however, studies estimate that 11–50% of patients with SUD meet the criteria for comorbid PTSD.^3^ Less information is available on the rates of PTSD in patients with opioid use disorder (OUD); however, they have been reported to be among the highest (41% lifetime, 33% current).^4^ Fluctuations in PTSD symptoms are risk factors for opioid use in patients receiving treatment,^5^ and may be associated with more intense cravings^6,7^ and increased psychological distress.^8^ Comorbid PTSD and SUD is also associated with higher rates of other psychiatric and physical comorbidities,^9^ fewer social supports and higher rates of unemployment.^7^ Patients with comorbid SUD and PTSD may have worse treatment outcomes,^10^ and are at higher risk for suicide attempts, relapses to substance use and treatment drop-out.^6,10^ Despite these findings, there exists limited formal protocol and guidance to address these co-occurring issues.^11^ First-line treatments for PTSD include psychotherapies such as trauma-focused cognitive–behavioural therapy, cognitive processing therapy, prolonged exposure, and eye movement desensitisation and reprocessing therapy; first-line medication treatments include antidepressant treatment with selective serotonin reuptake inhibitors or serotonin–norepinephrine reuptake inhibitors, and targeted symptom treatments have been recommended such as the use of prazosin for nightmares.^12,13^ No specific guidelines for the treatment of PTSD in patients with OUD have been published, and the availability of these first-line psychotherapies in addiction treatment settings is limited.^14,15^ Acknowledging the high prevalence of PTSD in patients with SUDs and the associated negative impact on treatment, we sought to further examine traumatic events and PTSD in a cohort of patients receiving opioid agonist treatment for OUD. The objectives of this study were to (a) assess the prevalence of traumatic events, with and without current PTSD, in a cohort of patients receiving treatment for OUD; (b) describe demographic and clinical characteristics of patients with history of traumatic events or PTSD and (c) explore the association between traumatic events and PTSD and psychosocial and clinical outcomes during OUD treatment.

## Method

We used prospective data from the Genetics of Opioid Addiction (GENOA) study conducted in Southern Ontario, Canada, between June 2011 and April 2017. The GENOA study sought to explore biological, psychological and social factors associated with treatment outcomes for patients with OUD. Data were collected from participants older than 18 years of age, who were receiving methadone maintenance treatment (MMT) for a diagnosis of OUD (as per the DSM-IV-TR).^16^ Methadone remains the most commonly prescribed pharmacological treatment for OUD in Canada, and at the time that this study was conducted, buprenorphine/naloxone was not covered through the Ontario provincial drug plan. The diagnosis of OUD was established by treating physicians and is an eligibility criterion for clinical follow-up at the clinics included in this study. Participants could be enrolled in MMT for any length of time before recruitment (median 3 years) and participants with polysubstance use or co-occurring SUDs (in addition to OUD) were included. No other inclusion or exclusion criteria were applied, to increase the generalisability of the sample. Participants were recruited from 20 community-based out-patient clinics that are run centrally through a single management team and follow the same clinical protocols. Patients enrolled in participating clinics have access to a multidisciplinary team of health professionals, including addiction physicians, nurses, pharmacists and staff experienced in addiction treatment. They receive medical examination and laboratory tests as indicated, as well as supervised urine testing. All patients included in the present study were receiving methadone treatment but could be prescribed any other medications by their clinic physician or other physicians involved in their care. Patients have access to harm reduction services, including naloxone kits and needle exchange programmes, as well as supports to connect with community agencies to provide services including counselling, housing support, job banks and primary care. No formal psychotherapy treatments are offered in the clinics.

The authors assert that all procedures contributing to this work comply with the ethical standards of the relevant national and institutional committees on human experimentation and with the Helsinki Declaration of 1975, as revised in 2008. All procedures involving human patients were approved by the Hamilton Integrated Research Ethics Board (project identifier 11-056), and all study procedures were conducted in accordance with their ethical guidelines. Written and verbal informed consent was obtained from every participant. Our study methods and findings are reported in accordance with the Strengthening the Reporting of Observational Studies in Epidemiology (STROBE) guidelines.^17^

**Fig. 1:**
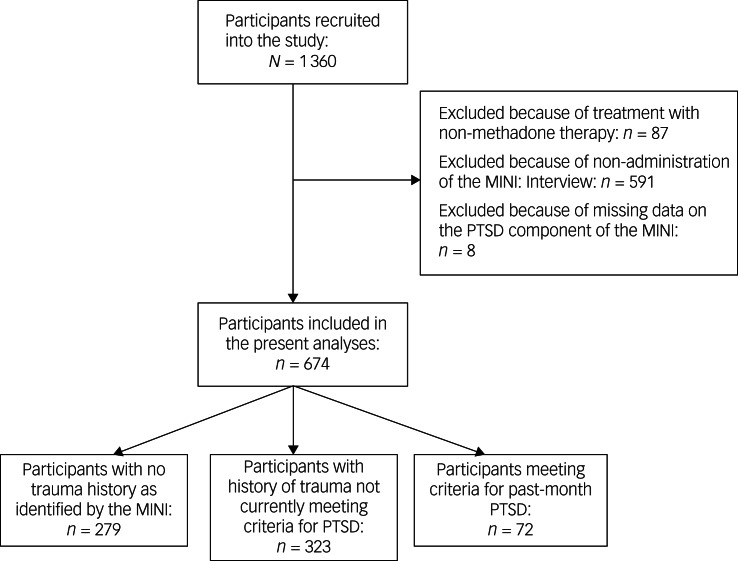
Study flow diagram. MINI, Mini-International Neuropsychiatric Interview; PTSD, post-traumatic stress disorder.

Participants completed face-to-face interviews at the time of study entry, to obtain information on demographic characteristics, medical and psychiatric history, medications, methadone dose and length of time in treatment. The first 680 participants consecutively recruited into the study competed the Mini-International Neuropsychiatric Interview (MINI) version 6.0,^18^ administered by trained interviewers at the time of study entry. Use of the MINI was subsequently discontinued in the study because of the time burden of administration. Therefore, inclusion in the present study's analyses was limited to those participants who were administered the MINI (Fig. 1, study flow diagram). Included in the MINI diagnostic interview is a screen for lifetime history of traumatic events, elicited through the following question: ‘Have you ever experienced or witnessed or had to deal with an extremely traumatic event that included actual or threated death or serious injury to you or someone else? Examples of traumatic events include: serious accidents, sexual or physical assault, a terrorist attack, being held hostage, kidnapping, fire, discovering a body, war, or natural disaster, witnessing the violent or sudden death of someone close to you, or a life-threatening illness’. This screening question is based on DSM-IV-TR criterion A for the diagnosis of PTSD.^16^ Subsequently, participants were asked about symptoms of PTSD as per the DSM-IV-TR,^16^ to determine a past-month diagnosis of PTSD. For the purposes of this study, participants who screen positive for past-month PTSD are considered as having ‘current PTSD’. Individuals who report experiencing a traumatic event but do not meet past-month criteria for PTSD are categorised as having a history of ‘traumatic events without current PTSD’. Individuals who deny lifetime history of traumatic events are considered to have had ‘no trauma’. These three groups are considered mutually exclusive. Finally, participants were also asked to report past-month suicidal ideation and past-month suicide attempts in the MINI assessment.^18^

Participants were followed in the study for 12 months. Use of opioids, cocaine and benzodiazepines were measured with urine drug screen tests, generally administered weekly, following clinical protocol. Urine testing for amphetamines and cannabis was conducted inconsistently, and is therefore not included in the present analyses. Urine toxicology screening was conducted with the iMDx Prep assay.^19^ The percentage of positive urine drug screens was calculated for each patient, for each substance. Ongoing opioid use was defined as at least one opioid-positive urine drug screen during the study.

### Statistical analyses

Analyses were conducted with Stata version 15.1 for Mac (StataCorp, USA). Our first objective was to determine the prevalence of traumatic events and current PTSD in our study cohort, and our second objective was to describe the demographic and clinical characteristics of these groups. We present descriptive statistics based on group status (i.e. history of traumatic event without current PTSD, current PTSD, no trauma). Continuous variables were summarised as means and s.d. if normally distributed, and as medians with interquartile range (IQR) for skewed data. IQR represents the difference between Q3 and Q1 and is defined as IQR = Q3 − Q1. Categorical variables were summarised with frequencies and percentages. Our third objective was to explore the association between trauma history and demographic characteristics and treatment outcomes. We constructed a multinomial logistic regression model with group status (history of traumatic event without current PTSD, current PTSD or no traumatic events or PTSD) as the dependent variable. Covariates included in the model were selected based on previous literature suggesting associations with trauma history or medication-assisted treatment outcome: gender,^20^ methadone dose,^21^ employment status^7^ and marital status.^7^ We also included age at onset of opioid use, length of time in treatment, ongoing opioid use and suicidal ideation as covariates in the model, to explore their association with group status. Results are represented as odds ratios with 95% confidence intervals.

## Results

Altogether, 1360 unique participants were recruited into the study and 674 participants who were administered the MINI were included in the present analyses (Fig. 1). Forty-one per cent of participants were identified to have no trauma history (*n* = 279), and 48% of participants reported a lifetime history of traumatic events, without meeting criteria for PTSD in the past month (*n* = 323). Eleven per cent of participants met past-month MINI criteria for PTSD (*n* = 72).

Table 1 details sociodemographic and clinical characteristics of participants, by group. Among participants with PTSD, 63% were female, compared with 44% in the other two groups. The mean age of participants in all three groups was approximately 39 years. Among participants with no history of trauma, 35% were married, compared with 28% of participants with history of traumatic events but no PTSD and 21% of participants with PTSD. Unemployment rate was highest for participants with PTSD (83%), followed by participants with traumatic events without PTSD (71%) and participants with neither (56%). Participants were enrolled in treatment for a median of 3 years (IQR = 5). Participants with PTSD had the highest median methadone dose at 84.5 mg/day (IQR = 52.5), compared with 70 mg/day in the other two groups.

**Table 1:** Baseline demographics and clinical information (*N* = 674)

Characteristic	Total sample (*N* = 674)	No trauma (*n* = 279)	Traumatic events without current PTSD (*n* = 323)	Current PTSD (*n* = 72)
Sociodemographic				
Age, years, mean (s.d.)	38.8 (11.1)	38.7 (11.2)	38.9 (11.2)	38.7 (10.7)
Female gender, *n* (%)	310 (46%)	124 (44.4%)	141 (43.7%)	45 (62.5%)
Married or common law, *n* (%)	202 (30%)	97 (34.8%)	90 (27.9%)	15 (20.8%)
Unemployment, *n* (%)	444 (65.9%)	155 (55.6%)	229 (70.9%)	60 (83.3%)
Clinical				
Age at onset of opioid use (years),^a^ mean (s.d.)	25.5 (8.76)	26.9 (9.3)	24.6 (8.2)	23.3 (8.1)
Methadone dose, mg/day, median (IQR)	70 (55)	70 (55)	70 (62)	84.5 (52.5)
Duration in treatment, years, median (IQR)	3 (5)	2.8 (4.3)	3 (6)	2.9 (6)
Antidepressant prescription, *n* (%)	187 (27.7%)	68 (24.4%)	91 (28.2%)	28 (38.9%)
Prazosin prescription, *n* (%)	1	0	1 (0.31%)	0
Benzodiazepine prescription, *n* (%)	158 (23.4%)	49 (17.6%)	83 (25.7%)	26 (36.1%)
Self-reported past-month suicidal ideation, *n* (%)	66/666 (9.9%)	20/276 (7.3%)	32/319 (10%)	14/71 (19.7%)
Self-reported past-month suicide attempt, *n* (%)	15/658 (2.3%)	5/275 (1.8%)	8/312 (2.6%)	2/71 (2.8%)
Ongoing use of opioids (non-abstinence), *n* (%)	515 (76.4%)	201 (72%)	257 (79.6%)	57 (79.2%)
Percentage of opioid-positive urine drug screens among non-abstainers, mean (s.d.)	23.2 (27.1)	23.8 (26.7)	24.3 (28.1)	16 (23)
Use of other substances, *n* (%)				
Non-prescribed benzodiazepines^b^	172 (25.5%)	68 (24.4%)	87 (26.9%)	17 (23.6%)
Cocaine	250 (37.1%)	101 (36.2%)	125 (38.7%)	24 (33.3%)
Percentage of positive urine drug screens among those who use other substances, median (IQR)				
Non-prescribed benzodiazepines^b^	27.9 (52.8)	30 (53.2)	26.8 (53.3)	31.6 (54.7)
Cocaine	26.7 (56.7)	29.4 (71.4)	24 (55.2)	25.8 (56.3)

PTSD, post-traumatic stress disorder; IQR, interquartile range.

a. Data available for 653 participants: 274 participants in no trauma group, 308 participants in the traumatic events without current PTSD group and 71 participants in the PTSD group.

b. Non-prescribed benzodiazepine use was defined as benzodiazepines use detected on urine drug screening with no benzodiazepine prescription in the clinic medical chart.

Antidepressant medications were prescribed for 39% of participants with PTSD, 28% of participants with past traumatic events without current PTSD and 24% of participants with no history of trauma (Table 1). Just one participant in the entire study sample was prescribed prazosin. The prevalence of benzodiazepine prescription was 36% in participants with PTSD, 26% in participants with traumatic events but no PTSD and 18% in participants with no history of trauma. Past-month suicidal ideation was reported by nearly 20% of participants with current PTSD; higher than in the other two groups, in which 7% and 10% of participants reported suicidal ideation, respectively. Reports of past-month suicide attempts were lower at 1.8% in participants with no trauma history, 2.6% in participants with traumatic events but no PTSD and 2.8% in participants with current PTSD. Seventy per cent of participants with no trauma history had ongoing opioid use as evidenced by at least one opioid-positive urine drug screen, compared with 80% of participants with traumatic events and no PTSD and 79% of participants with PTSD. Non-prescribed benzodiazepine use was identified in about 24% of both participants with PTSD and those with no trauma history, and in about 27% of participants reporting traumatic events and no current PTSD. Cocaine use was identified in 33% of participants with PTSD, 39% of participants with traumatic events and no current PTSD and 36% of participants with no trauma history.

We present the results of multinomial regression analysis in Table 2. Participants with PTSD were more likely to be female than those with no trauma history (odds ratio 2.13, 95% CI 1.20–3.76, *P* = 0.009). Compared with participants with no history of trauma, both those with traumatic events without PTSD and those with PTSD were less likely to be employed (odds ratio 0.53, 95% CI 0.37–0.75, *P* < 0.001; and odds ratio 0.31, 95% CI 0.16–0.61, *P* = 0.001, respectively). Similarly, compared with participants with no history of trauma, having traumatic events without PTSD or with PTSD were both associated with younger age at onset of opioid use (odds ratio 0.97, 95% CI 0.95–0.99, *P* = 0.002; and odds ratio 0.95, 95% CI 0.92–0.98, *P* = 0.004, respectively). Participants with PTSD were also less likely to be married than participants with no trauma history (odds ratio 0.51, 95% CI 0.26–0.99, *P* = 0.046). There were no significant associations between trauma history and duration in MMT (*P* = 0.204 for patients with trauma without current PTSD and *P* = 0.691 for patients with PTSD). No association between ongoing opioid use and trauma status was seen (Table 2); however, participants with PTSD were more likely to report past-month suicidal ideation (odds ratio 2.29, 95% CI 1.04–5.01, *P* = 0.039).

**Table 2:** Multivariable model of demographic and clinical factors associated with trauma history

Trauma history	Covariate	Odds ratio	95% CI	*P-*value
No trauma	(reference group)			
Traumatic event without current PTSD	Male gender	[Reference]
	Female gender	0.91	0.64–1.28	0.577
	Methadone dose	1.0	1.0–1.0	0.872
	Years in MMT^a^	1.03	0.98–1.07	0.204
	Employed	0.53	0.37–0.75	<0.001
	Married or common law	0.74	0.51–1.06	0.098
	Age at onset of opioid use^b^	0.97	0.95–0.99	0.002
	Opioid abstinence	[Reference]
	Opioid use	1.41	0.95–2.10	0.090
	Suicidal ideation	1.12	0.61–2.06	0.710
Current PTSD	Male gender	[Reference]
	Female gender	2.13	1.20–3.76	0.009
	Methadone dose	1.0	1.0–1.0	0.264
	Years in MMT^a^	1.01	0.95–1.08	0.691
	Employed	0.31	0.16–0.61	0.001
	Married or common law	0.51	0.26–0.99	0.046
	Age at onset of opioid use^b^	0.95	0.92–0.98	0.004
	Opioid abstinence	[Reference]
	Opioid use	1.43	0.73–2.81	0.294
	Suicidal ideation	2.29	1.04–5.01	0.039

PTSD, post-traumatic stress disorder; MMT, methadone maintenance treatment.

a. Scaled for every 1-year increase in length of time in MMT.

b. Scaled for every 1-year increase in age at onset of opioid use.

## Discussion

In our cohort of patients receiving MMT for OUD, we identified an 11% past-month prevalence of PTSD. An additional 48% of participants reported a lifetime history of traumatic events without current PTSD. Therefore, for most patients treated for OUD, a consideration of the presence of trauma symptoms, their impact on substance use course and need for treatment is warranted. Factors associated with PTSD included female gender, unemployment, single marital status, younger age at onset of opioid use, and suicidal ideation; factors associated with a history of trauma without current PTSD included unemployment and younger age at onset of opioid use.

We found important differences in sociodemographic characteristics among patients with and without trauma. Patients with past traumatic events, with or without current PTSD, had a younger age at onset of opioid use. Early identification and management of trauma and PTSD could potentially affect the development or progression of OUD. Although outside of the scope of the present study, the possibility that the identification and management of PTSD may affect OUD outcomes is an area that requires further research. Patients with comorbid OUD and PTSD were also more likely to be unemployed and single when compared with patients with no trauma history. These individuals may require different psychosocial supports within their treatment programmes.^22^

### Psychiatric medications

Our examination of the psychiatric medications prescribed for patients with comorbid PTSD revealed several areas of concern. Only 39% were receiving treatment with antidepressants, which are evidence-based treatments for PTSD.^12,13^ A limitation of this study is the lack of information on concurrent psychotherapeutic treatments; however, no recommended psychological treatments for PTSD are available through the clinics included in this study. It is also important to consider that selective serotonin reuptake inhibitors can be used for the treatment of multiple psychiatric diagnoses and their prescription does not imply treatment for PTSD. Furthermore, only one participant was receiving prazosin, a medication indicated in PTSD for reducing the severity and frequency of nightmares.^12^ Sleep disturbance was reported by 92% of participants with PTSD and re-experiencing symptoms (including dreams, intense recollections or flashbacks) was reported by 100%. Previous studies have reported the prevalence of nightmares in PTSD to be 50–70%, whereas the prevalence of sleep disturbance is reported in 40–50% of patients.^23^ Addressing chronic nightmares is of utmost importance as they result in decreased psychological and physical health and may amplify other PTSD symptoms, including suicidal ideation.^24^ Our findings suggest that more can be done to offer evidence-based treatments for PTSD in patients with this comorbidity.

Additionally, 36% of patients with comorbid PTSD were prescribed benzodiazepines, which are neither a recommended treatment for PTSD^12,13^ nor recommended for use in patients receiving opioid agonist treatment, because of the risks of respiratory suppression.^11^ It is possible that benzodiazepines are being prescribed to alleviate symptoms of PTSD, including anxiety, insomnia and irritability.^25^ Although studies estimate that benzodiazepines are prescribed to approximately 30–75% of patients with PTSD, a recent systematic review has concluded that benzodiazepines are ineffective for this purpose, with the risks outweighing the benefits.^25^ The concomitant use of benzodiazepines and opioids increases the risk of respiratory suppression and death, and has not been shown to improve treatment retention.^11^ Our findings may inspire reflection among clinicians treating this patient population, and prompt further research into the pharmacological treatment of patients with comorbid OUD and PTSD.

### Treatment outcomes

We did not identify significant differences in the length of time in treatment, or in ongoing opioid use based on trauma history. In fact, many participants (>70% of the total study sample) had ongoing opioid use (defined as at least one opioid-positive urine drug screen during the study), regardless of trauma history. Severity of opioid use, as measured by the percentage of opioid-positive urine drug screens, also was not associated with trauma status (data not shown). Other substances examined, including cocaine and non-prescribed benzodiazepines, were also used at comparable rates between groups. Our study did not examine drop-out from treatment because the median length of time in treatment was quite long, at 3 years (IQR = 5). A previous study of patients enrolled in an out-patient buprenorphine treatment programme found that individuals with childhood trauma were more likely to drop out of treatment;^26^ however, two studies of patients enrolled in MMT found no significant association between the diagnosis of PTSD and 3-month^27^ or 1-year retention in treatment.^28^ Future studies that further examine the impact of trauma and PTSD on retention in treatment in patients newly starting opioid agonist treatment may be helpful.

The psychological outcomes of patients with comorbid OUD and PTSD are also important to consider. A previous study including patients with comorbid SUDs and PTSD identified these patients as having greater difficulty with coping skills and adaptive cognitions, resulting in increased psychological distress.^8^ In our study, nearly 20% of participants with PTSD reported suicidal ideation in the past month, and nearly 3% reported suicide attempts in the past month. Suicidal ideation and suicide attempts were reported at lower rates in the other groups, with the PTSD group more than twice as likely to report suicidal ideation, adjusting for other covariates. This finding highlights that PTSD is not a benign comorbidity in OUD. Patients with OUD and suicidal ideation are at risk for high-lethality overdoses.^29^ Taken together, these findings suggest that some patients with trauma history or PTSD may have comparable substance use outcomes in treatment, but have psychiatric and psychosocial differences that require attention. Our findings are consistent with two previous studies that have found good substance use outcomes in opioid agonist treatment, but poor or unchanged psychiatric outcomes for comorbid PTSD,^30,31^ suggesting that additional services are required for these patients.

### Implications

Improving treatment of PTSD in patients with SUD is critical;^30^ studies have demonstrated that remitted PTSD is associated with decreased substance use,^32^ whereas a lack of improvement in PTSD symptoms is associated with increased substance use.^33^ Patients with SUD, regardless of comorbidity, are often solely treated for their substance use upon first clinical contact.^34^ After achieving sobriety, these patients may then be referred for PTSD treatment.^34^ However, this model of sequential treatment in which OUD is treated before the initiation of PTSD treatment may be ineffective and inconsistent with research, which supports an integrated approach.^10,34^ A number of different treatment models have found success with integrated treatment programmes.^10^ The basis for these integrated programmes is that improvement in PTSD symptoms is more likely to attenuate SUD symptoms, but the reverse does not result in the same effect.^10^ Despite these findings, the uptake of such programmes has been low, and more research is needed to identify potential barriers that prevent implementation of integrated treatment.^10^

### Strengths and limitations

Strengths of this study include its sample size and use of the validated MINI tool to identify trauma comorbidity. Healthy patient and volunteer biases may limit the findings of this study such that patients with trauma comorbidity may have been less likely to participate; therefore, we likely underestimate the prevalence of traumatic events and PTSD.

Through the use of broad inclusion criteria and multi-site sampling, we increase the generalisability of our findings. Participants included in this study were recruited from 20 different community-based out-patient addictions clinics in southern Ontario. The Canadian Agency for Drugs and Technologies in Health published an environmental scan of programmes for the treatment of opioid addiction in Canada in September 2019. Findings from this report suggest that most services accept patients who have a DSM-diagnosed OUD with not many other restrictions.^35^ This suggests that the inclusion criteria for our study are appropriate and reflective of individuals who would be eligible to enter treatment in the general population. Public Health Ontario data from 2017 indicate that the population of patients receiving opioid agonist therapy in Ontario is comparable, in terms of age and gender, with the patients recruited in our study: 64% of patients treated in Ontario were 25–44 years of age, and 63% of patients treated were male.^36^ As in all studies, there are possible limitations to generalisability that must be considered. For example, all patients in this study were receiving MMT, thus findings may not be applicable to individuals with OUD who are not enrolled in treatment. Further, all patients in this study were agreeable to being recruited and were able to complete the assessments, therefore there is a possibility of volunteer bias. Although we use urine drug screen results to measure opioid use, we are unable to quantify the extent or severity of OUD based on the extent or amount of opioid used. Finally, in Ontario, and more generally in Canada, opioid agonist treatment takes on a harm reduction role, meaning that patients are not discharged from treatment as a result of relapse to opioid or other substance use. Our results may not be generalisable to settings in which ongoing treatment is contingent on abstinence from opioids or other substances.

As this is an observational study, we cannot make conclusions about causality, and there is risk of unmeasured confounding in our analyses. Future studies that examine the longitudinal course of trauma and PTSD in this population will be beneficial, as will studies that shed light on patient goals and treatment priorities in the context of trauma and substance use comorbidity.

In conclusion, patients with OUD and history of trauma or comorbid PTSD differ from patients without trauma comorbidity on a number of clinical and psychosocial factors. We found that patients with trauma history or PTSD may have comparable substance use outcomes in treatment of OUD, but have psychiatric and psychosocial differences that require attention. We also identified that the psychopharmacological treatment of PTSD in patients receiving opioid agonist treatment is an area requiring attention. Our findings emphasise the relevance of better integrating addictions and mental health services for this population.

## Data Availability

The data that support the findings of this study are available from the corresponding author, Z.S., upon reasonable request.

## Supplementary material

For supplementary material accompanying this paper visit http://dx.doi.org/10.1192/bjo.2021.971. click here to view supplementary material
